# Effects of α lipoic acid combined with olmesartan medoxomil on blood glucose and oxidation indicators in patients with diabetic nephropathy: A protocol for a parallel, randomized, double-blind, controlled clinical trial

**DOI:** 10.1097/MD.0000000000029080

**Published:** 2022-05-06

**Authors:** Shengfu Jiao, Yuxia Dong, Xiang Chang, Yanan Wu, Haifeng Li

**Affiliations:** a Wuwei Hospital of Traditional Chinese Medicine, Wuwei, Gansu Province, China; b Wuwei People's Hospital, Wuwei, Gansu Province, China.

**Keywords:** α lipoic acid, diabetic nephropathy, olmesartan medoxomil, protocol, randomized controlled trials

## Abstract

**Background:**

Diabetic nephropathy (DN) is a common microvascular complication of diabetes, which poses a serious threat to the health and life of patients. There is evidence that both α lipoic acid and olmesartan medoxomil have positive effects in the treatment of DN, but whether the 2 have synergistic effects and the effects on blood glucose and oxidation indicators are controversial.

**Methods:**

This is a prospective parallel, randomized, double-blind, placebo-controlled trial to study the effects of α lipoic acid in combination with olmesartan medoxomil on blood glucose and oxidation indicators in patients with DN. Participants will be randomly assigned to a treatment group, which will receive α lipoic acid dispersive tablets combined with olmesartan medoxomil tablets, or a control group, which will receive olmesartan medoxomil tablets combined with placebo for 4 weeks, followed up for 12 weeks. Observation indicators include: glycemic indicators [fasting blood glucose, 2 hours postprandial blood glucose and glycosylated hemoglobin], the oxidation indicators [serum glutathione, superoxide dismutase, malondialdehyde, 8-hydroxydeox-yguanosine], and adverse reactions. Finally, SPASS 22.0 software will be used for statistical analysis of the data.

**Discussion:**

This study will evaluate the effects of α lipoic acid combined with olmesartan medoxomil on blood glucose and oxidation indicators in patients with DN. The results of this study will provide a reference for the clinical use of α lipoic acid combined with olmesartan medoxomil in the treatment of DN.

**Trial registration:**

OSF Registration number: DOI 10.17605/OSF.IO/VJWXS

## 1 Introduction

The incidence of diabetes is increasing year by year. According to the statistics of the International Diabetes Federation, there are about 415 million people with diabetes in the world at present, and the number of diabetes patients is expected to reach 642 million by 2040.^[1]^ Diabetic nephropathy (DN) is a renal structural change and abnormal function caused by chronic diabetic microangiopathy. About 30% to 40% of diabetic patients will develop into DN.^[2]^ If DN is not treated in time, it will lead to renal failure and ultimately rely on kidney dialysis to maintain life.^[3]^ Some studies have found that the 5-year survival rate of patients with end-stage renal disease is less than 20%.^[4]^

The generation of DN is not only related to glucose metabolism, but also closely related to oxidative stress.^[5]^ The main mechanism of DN is oxidative stress response caused by inflammation caused by hyperglycemia,^[6]^ and experimental studies have also confirmed the role of oxidative stress in the occurrence and progression of DN.^[7]^ At present, the treatment of DN mainly focuses on controlling blood glucose, improving oxidative stress response and controlling proteinuria. Early treatment to avoid further kidney damage is particularly important for DN patients.^[8–10]^

α lipoic acid is a strong antioxidant, which can improve the antioxidant capacity of the body and inhibit lipid peroxidation in vivo, thus protecting the vascular endothelium and preventing and alleviating the occurrence and progression of DN.^[11]^ Clinical studies have confirmed that lipoic acid injection can effectively reduce the level of oxidative stress, blood glucose and blood lipids in DN patients, and improve the renal function of patients.^[4]^ Olmesartan medoxomil is an angiotensin II receptor antagonist, which can delay glomerulosclerosis, reduce the release of inflammatory factors, prevent and reduce the production of urinary protein, and has a protective effect on the kidney.^[12]^ At present, some scholars recommend the combination of the 2 drugs, and believe that the 2 drugs have a synergistic effect. Some clinical studies have also confirmed that α lipoic acid combined with olmesartan medoxomil can improve the blood glucose level of DN patients, reduce blood glucose fluctuations, relieve the body's oxidative response, and reduce inflammation.^[13]^ However, there is still a lack of rigorous randomized controlled studies to verify these conclusions. Therefore, this study will investigate the effects of α lipoic acid combined with olmesartan medoxomil on blood glucose and oxidation indicators in DN patients through a prospective parallel, randomized, double-blind, placebo-controlled trial.

## 2 Materials and methods

### 2.1 Study design

**Figure 1:**
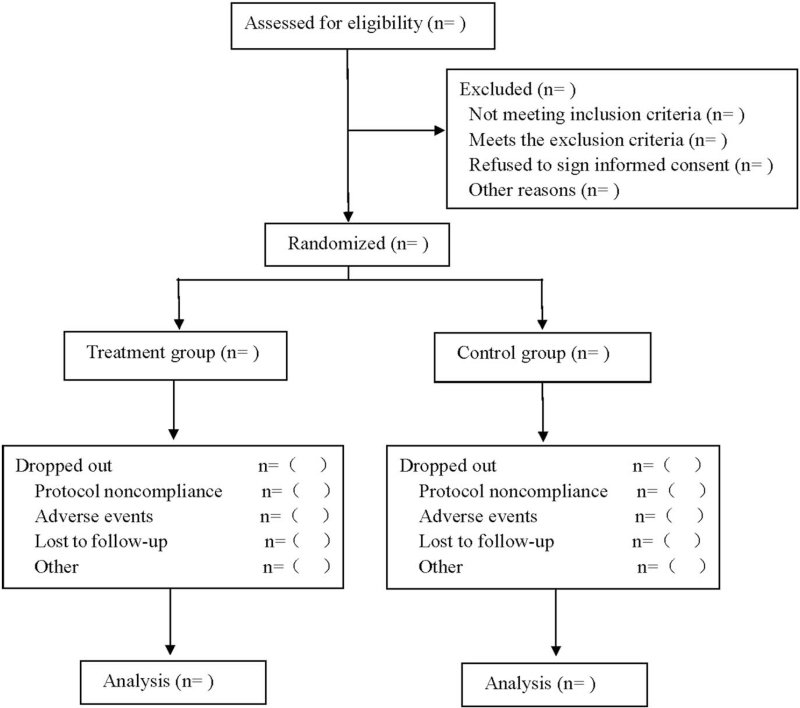
Flow diagram.

This is a prospective, randomized, double-blind, placebo-controlled study of α lipoic acid in combination with olmesartan medoxomil on blood glucose and oxidation indicators in patients with DN. Participants will be randomly assigned to either a treatment group receiving olmesartan medoxomil tablets in combination with alpha lipoic acid dispersive tablets or a control group receiving olmesartan medoxomil tablets in combination with placebo for 4 weeks and followed up for 12 weeks, see Figure 1 for flow diagram and Table 1 for study schedule. This protocol followed the latest Consolidated Standards of Reporting Trials and Standard Protocol Items: Recommendations for Interventional Trials 2013 statement.

**Table 1:** Study schedule.

	Screening period	Treatment period		Follow-up		
Stage Project	Baseline	2-week	4-week	4-week	8-week	12-week
Record fill	√					
Fulfill inclusion criteria and exclusion criteria	√					
Sign informed consent	√					
Random allocation	√					
Treatment	√	√	√			
Effectiveness observation						
Fasting blood glucose (FBG)	√		√			√
2 h postprandial blood glucose	√		√			√
Glycosylated hemoglobin (HbA1c)	√		√			√
Glutathione (GSH)	√		√			√
Superoxide dismutase (SOD)	√		√			√
Malondialdehyde (MDA)	√		√			√
8-hydroxydeox-yguanosine (8-OHdG)	√		√			√
Safety evaluation						
Blood test and urinalysis	√		√			√
Liver and kidney function	√		√			√
Record of adverse event		√	√	√	√	√

### 2.2 Ethics and registration

This Research protocol will be conducted in accordance with the Declaration of Helsinki and Ethical Guidelines for Clinical Research. This study has been approved by our Clinical Research Ethics Committee and registered with the Open Science Framework (registration number: DOI 10.17605/OSF.IO/VJWXS). Before randomization, all patients will be required to sign an informed consent form, giving them the option of continuing the trial at any time.

### 2.3 Patients

#### 2.3.1 Diagnosis basis

The diagnostic criteria for DN refer to the report from an American Diabetes Association Consensus Conference in 2014.^[14]^

#### 2.3.2 Inclusion criteria

1. Meet the diagnostic criteria of DN;
2. Age ≥18 and ≤70 years old;
3. Subjects agree to participate in this study and sign informed consent.

#### 2.3.3 Exclusion criteria

1. primary kidney disease or renal failure caused by other diseases;
2. complicated with functional impairment of heart, brain, liver, and other important organs;
3. patients with acute or chronic infection;
4. women who are pregnant, preparing for pregnancy or breastfeeding;
5. patients who are allergic to drugs used in the study;
6. patients have serious mental illness or cannot cooperate with the researcher;
7. those who have participated in or are currently participating in other clinical trials within the past 1 month.

For patients who drop out of the trial or lost to follow-up, researchers should actively take measures to complete the last test as far as possible, so as to analyze the efficacy and safety of the test and take corresponding treatment measures. All cases of detachment should be recorded on the case report form (CRF), fill in the cause of case shedding.

### 2.4 Sample size

The sample size is estimated based on the mean and standard deviation of fasting blood glucose scores after 4 weeks of treatment, which were 5.97 ± 1.82 in the treatment group and 7.28 ± 2.34 in the control group referring to the pre-experiment results. Set α = 0.025, unilateral test, β = 0.20. The PASS15.0 software calculated those 42 participants were required for each group, with an estimated drop-out rate of 10%, and 47 patients would be enrolled in each group.

### 2.5 Randomization and blinding

We will use a centralized network-based randomization tool to randomly assign patients who meet the criteria to treatment or control at a ratio of 1:1. Random sequences will be generated using SAS 9.3 software (SAS Institute, Cary, NC) by independent statisticians not involved in trial implementation or statistical analysis. The research assistant will enter patient information on a tablet computer and be assigned a random number to complete the random assignment. The outcome of the assignment is unknown to the patients and principal investigator throughout the study.

### 2.6 Interventions

All patients will receive the same basic care, including a low-salt, low-fat diabetic diet, moderate exercise, and control of blood sugar and blood pressure.^[13]^ All of these basic treatments will be detailed on the patient's medical record and CRF.

Treatment group: α lipoic acid dispersive tablets (Tonghe Pharmaceutical Co., LTD., Jiangsu, China, 0.1 g/ tablet), 2 tablets each time, 3 times a day, orally; Olmesartan medoxomil tablet (Wansheng Pharmaceutical Co., LTD., Beijing, China, 20 mg/ tablet); 1 tablet each time, once a day, orally. It lasts for 4 weeks.

Control group: Olmesartan medoxomil tablets (Wansheng Pharmaceutical Co., LTD., Beijing, China, 20 mg/ tablet); 1 tablet each time, once a day, orally; Placebo (mainly composed of starch and dextrin), which has the same shape, size and color as alpha lipoic acid dispersive tablets, prepared and provided by Jiangsu Famai Sheng Medical Technology Co., LTD., China. 2 tablets each time, 3 times a day, orally; It lasts for 4 weeks.

### 2.7 Outcomes

1. Blood glucose indicators: fasting venous blood of the patients will be collected early in the morning before and after the treatment, and automatic biochemical analyzer (HITEC-7100, Japan) will be used to measure the blood glucose indicators of the patients, including fasting blood glucose, 2 hours postprandial blood glucose and glycosylated hemoglobin.
2. Oxidation indicators: we will use the radioimmunoprecipitation kit (Hengyuan Biotechnology Co., LTD., Shanghai, China) to detect serum glutathione, superoxide dismutase, malondialdehyde, and 8-hydroxydeox-yguanosine.
  Two research assistants will be responsible for data collection. During the follow-up period, the research assistants will collect data information based on the above indicators at week 4 and week 12 after treatment.

### 2.8 Safety evaluation

Patients’ blood routine, urine routine, liver function, kidney function and electrocardiogram will be tested at baseline and after treatment to assess the safety of treatment. Details of all adverse events will be recorded in the CRF, including the time, extent and duration of occurrence, suspected reasons, effective measures and outcomes. After treatment, the incidence of adverse reactions in the 2 groups will be analyzed.

### 2.9 Data management and quality control

Any modification or change to the protocol will be re-approved through the formal procedures of our Ethics Committee. Independent clinical research assistants will periodically review research progress. Study data will be collected and recorded in CRF by trained investigators. To ensure data reliability, personal information about potential and registered participants will be collected, shared and kept in a separate repository to protect confidentiality before, during and after the trial. Access to the database will be restricted to the researchers in the research team. Participants’ information will not be disclosed or shared without their written permission.

### 2.10 Statistical analysis

The collected data will be statistically analyzed by SPSS 22.0 software. Chi-Squared test will be used for counting data. Mean ± standard deviation ($\bar{x}$ ± S) will be used for measurement data, independent sample *T* test will be used for normal distribution, and Mann–Whitney *U* test will be used for skewness distribution. The difference is considered statistically significant when *P* < .05.

## 3 Discussion

The pathogenesis of diabetic nephropathy is complex, involving the disorder of glucose metabolism, abnormal hemodynamics and hemorheology. Genes, inflammation and oxidative stress are also important reasons for the progression of DN.^[15]^ Current studies have found that oxidative stress products are involved in various pathogenesis of DN,^[5]^ oxidative stress may be an important link or even a central link in the pathogenesis of DN, and inhibition of oxidative stress may become an important direction for the prevention and treatment of DN alone or in collaboration.^[16]^

α lipoic acid is the oxidized form of lipoic acid, which can reduce oxidative stress levels by scavenging reactive oxygen species and free radicals. At present, α lipoic acid has been widely used in diabetes and its related complications, and has obtained positive efficacy.^[17,18]^ Ocamesartan ester is a highly selective angiotensin receptor blockers that binds to 2 sites of -OH and -COOH of AT1, which can prevent mesangial cell and fibrotic proliferation, prevent glomerulosclerosis, and protect the kidney.^[19]^ Some researchers believe that the combination of the 2 drugs can improve the clinical efficacy in DN patients,^[13]^ but high-quality clinical studies are lacking to confirm these conclusions. Therefore, this study is proposed to explore the effect of α lipoic acid injection combined with ocamesartan ester on blood glucose and oxidation indicators in DN patients using a standard randomized controlled experiment.

This study has some limitations: this study is a single-center randomized controlled study where the included population is regional and may affect the findings; Meanwhile, the follow-up time of this study is 3 months, and the effect of α lipoic acid combined with olmethartan ester can not be observed.

## Author contributions

**Conceptualization:** Shengfu Jiao and Xiang Chang.

**Data curation:** Shengfu Jiao and Yanan Wu.

**Formal analysis:** Shengfu Jiao and Haifeng Li.

**Funding acquisition:** Yuxia Dong.

**Software:** Haifeng Li and Xiang Chang.

**Supervision:** Yanan Wu and Xiang Chang.

**Writing – original draft:** Yuxia Dong and Yanan Wu.

**Writing – review & editing:** Yuxia Dong and Haifeng Li.
